# Acetyl-DL-leucine (Tanganil™) in three patients with advanced multiple system atrophy

**DOI:** 10.1186/s12883-025-04451-7

**Published:** 2025-10-02

**Authors:** Wolfgang H. Oertel, Martin T. Henrich, Elisabeth Sittig, Philipp T. Meyer, Michael Strupp, Annette Janzen, Fanni F. Geibl, Elisabeth Ruppert

**Affiliations:** 1 Department of Neurology, Philipps University of Marburg, Marburg, Germany; 2 Department of Psychiatry and Psychotherapy, Philipps University of Marburg, Marburg, Germany; 3 Department of Nuclear Medicine, Faculty of Medicine, Medical Center - University of Freiburg, University of Freiburg, Freiburg, Germany; 4 Department of Neurology, LMU University Hospital, LMU Munich, Munich, Germany; 5 Department of Neurology, CIRCSom (International Research Center for ChronoSomnology) and Sleep Disorders Center, University Hospital of Strasbourg, University of Strasbourg, Strasbourg, France

**Keywords:** N-acetyl-DL-leucine, MSA-C, Ataxia, REM sleep behavior disorder, Neurodegeneration, Adverse effects, Case report

## Abstract

**Background:**

Multiple system atrophy (MSA) is a rare, rapidly progressive alpha-synucleinopathy characterized by autonomic dysfunction, parkinsonism and marked cerebellar ataxia, with very limited treatment options. N-acetyl-DL-leucine (ADLL) (Tanganil™), a racemic derivative of leucine is usually well tolerated and has shown promise in improving cerebellar symptoms and reducing REM sleep behavior disorder (RBD), a common non-motor dream-sleep disorder feature of alpha-synucleinopathy. Given this dual potential, we treated three patients with advanced-stage MSA under compassionate use rules.

**Case presentations:**

Three patients with advanced-stage MSA and severe cerebellar symptoms—two of whom also had moderate RBD—were treated with ADLL (Tanganil^TM^), titrated to 5g/day over 10 days. While both patients with RBD reported self-assessed decrease of RBD symptoms within 2–3 weeks on the full dosage (approximately four weeks after treatment initiation), all three patients had major worsening of gait and balance during the same period, leading to sudden falls and overall health decline. These adverse events prompted early discontinuation of therapy. Gait improved within two weeks after discontinuation of therapy in two patients. In parallel, the RBD phenotype reoccurred. In the third, who had very advanced MSA and concurrent infections, gait only slowly improved over time and it remains unclear whether his worsening of truncal ataxia was attributable to ADLL (Tanganil^TM^), or whether it was related to his concurrent infections with slow recovery.

**Conclusions:**

While ADLL improved RBD, it was poorly tolerated in these three patients with advanced-stage MSA of predominantly cerebellar type. Further studies are needed to evaluate its safety and efficacy in different, preferably early, stages of MSA.

**Supplementary Information:**

The online version contains supplementary material available at 10.1186/s12883-025-04451-7.

## Background

Multiple system atrophy (MSA) is a rare alpha-synucleinopathy, characterised by rapidly evolving neurodegeneration, leading to severe disability within 5–6 years of symptom onset and death within about 10 years. Clinically, MSA presents with marked autonomic failure combined with parkinsonism, and/or a cerebellar syndrome. It is classified into MSA-parkinsonian type (MSA-P), MSA-cerebellar type (MSA-C) and MSA-P + C, depending on the predominant motor phenotype [1]. Current therapeutic approaches provide limited relief for MSA-related symptoms, underscoring an urgent need for novel treatment strategies and the development of disease-modifying therapies. In these case descriptions, we investigated the potential of ADLL (Tanganil™) [2–5] as a treatment for MSA, with a particular focus on cerebellar symptoms, given early observational studies with the racemate showing promise in treating patients with cerebellar ataxia, a key symptom in MSA patients [2, 6].

ADLL (N-acetyl-DL-leucine; Tanganil™) is the racemate of the modified, acetylated derivative of D- and L-leucine (Pierre Fabre, France), commonly used in France for the treatment of acute vertigo [7]. On the other hand, ALL (N-acetyl-L-leucine; Aqneursa™), the bioactive L-enantiomer, was approved in 2024 in the USA for the treatment of Niemann-Pick disease type C (NPC), a hereditary lysosomal storage disorder characterised by pronounced cerebellar symptoms.

Two prior case studies on the use of ADLL (Tanganil™) in patients with MSA-C have shown a lack of clear objective benefit on the cerebellar functions [8, 9]. In the first case series one patient with possible MSA-C (disease duration: 4 years) and three patients with probable MSA-C (disease duration: 1–4 years) were examined. Three patients reported subjective improvement. Median SARA (Scale for the Assessment and Rating of Ataxia) scores [10], assessed by three independent raters, improved by 1 and 2.5 points in two patients (including one who did not report subjective improvement) and worsened slightly by 0.5 points in the other two) [8]. In the second case study, nine patients with probable MSA-C were examined. After 4 weeks of treatment, five patients reported minimal improvement on the Patient Global Impression of Change (PGIC); of these, three reported continued minimal improvement after 12 weeks. Four patients opted to continue self-medication beyond 12 weeks. Two discontinued treatment, one due to lack of improvement, and the other due to side effects (muscle stiffness, numbness, and impaired gait), which subsided after discontinuation of ADLL. No other side effects were reported. Among the nine participants, only two required a walker for ambulation; the others did not require walking aids [9]. Another case series in patients with cerebellar ataxia also included two patients with MSA-C, both with a disease duration of three years. Four weeks of ADLL (Tanganil™) at 5 g per day led to improvements in their SARA scores of 1 and 0.5 points, respectively [11].

In respect to the alpha-synucleinopathy “isolated REM-sleep behaviour disorder (RBD) combined with hyposmia”, a phenotype considered to present a specific prodromal stage of Parkinson’s disease (PD) [12], ADLL has demonstrated marked efficacy in the treatment of the RBD symptoms in two patients [3]. In addition, ADLL-therapy dramatically reduced the “non-motor” symptom RBD in a patient with manifest advanced Parkinson’s Disease (PD) [4]. RBD is a clinical phenotype characterised by lack of atonia in REM-sleep, aggressive dream content and the enactment of these dreams ranging from talking to violent movements.

The pathophysiological mechanisms underlying the potential benefits of ADLL in patients with ataxia and/or RBD remain largely hypothetical. It is accepted, that ADLL and its bioactive enantiomer ALL improve lysosomal function [5, 13]. Further data suggest, that the L-enantiomer ALL could improve metabolic dysfunction by increasing ATP production and normalizing glucose/antioxidant metabolism [13]. In respect to cerebellar function ADLL may normalize vestibulo-cerebellar excitability [14] (see Supplementary discussion for details).

Given these descriptions of ADLL’s positive effects on RBD, despite the conflicting results on its effect on cerebellar symptoms, and based on the fact that the active L-enantiomer is not currently available for use anywhere in the world except the USA for NPC, we investigated the effect of ADLL in a compassionate-use setting for 3 patients with advanced-stage MSA, a disease frequently characterised by both cerebellar symptoms and RBD.

### Methodology

In these observational case descriptions (individual cases of off-label use; clinical trial number: not applicable), we selected three patients with clinically established, advanced-stage MSA [1]. All patients had previously been followed up at other movement disorder centres. The three MSA patients and their spouses contacted us on their own initiative as they had heard that the drug ADLL was effective for RBD [3]. All three patients lived between 350 and 500 km away from the Department of Neurology at the University Clinic in Marburg (UMR). The patients and spouses received detailed information about the compound ADLL (Tanganil™) including its lack of efficiency in the previously reported case series. Subsequently, the patients provided written informed consent to participate in an observational case description on the additional therapy with ADLL (Tanganil™) under compassionate use rules. The ADLL (Tanganil™) dosage was gradually increased over ten days to a final dose of 5 g/day, administered as ten 500 mg tablets per day: three tablets in the morning, two at noon, and five in the evening. This dosage is higher than the one used in cases of vertigo and corresponds to the regimen most commonly employed in ataxia, both in clinical practice and in previous studies. Assessments included the Unified Multiple System Atrophy Rating Scale (UMSARS) [15], daily self-ratings of the severity of RBD symptoms, and weekly self-ratings of the frequency of RBD symptoms, walking and falling symptoms and overall health state (see Supplementary Material for details). The daily self-assessment of RBD severity was completed using the following scale: 0 = no RBD symptoms as documented by patient and partner; 1 = speaking and/or slight movements (jerks); 2 = screaming, shouting or complex, non-aggressive movements; 3 = complex movements with risk of injury of herself/himself or partner; 4 = movements so severe that subject falls out of bed (Supplementary Fig. 1). Self-assessed health status was evaluated using a visual analogue scale, where 100 represented the best imaginable state and 0 the worst (Supplementary Fig. 2). Baseline neurological evaluations for patients #1 and #2 took place at the UMR conducted by a neurologist (AJ). At the end of the observational period, the neurological evaluation took place in the respective homes of the participants #2 and #3 and was conducted by a neurologist (ER). Owing to the geographical distance from their homes and their fragile health status, Patient #3 was unable to undergo the initially planned in-hospital baseline evaluation, and Patient 1 did not receive a final evaluation by the neurologist.

## Case presentations

Patient #1 and #2 suffered from MSA-C, while patient #3 had MSA-P + C. Patients #1 and #2 also had moderate RBD, scoring a maximum of 2 on a scale ranging from 0 (no symptom) to 4 (most severe symptoms). Regarding positive findings, both patients with RBD described relief of RBD symptoms after three to four weeks (Supplementary Fig. 1) following treatment initiation. However, the negative outcome was that all three patients had worsening of gait and balance symptoms after two to three weeks on the full dosage-approximately four weeks after treatment initiation-leading to snap-like falls in Patient #1. This deterioration was accompanied by a subjective sense of overall health decline (Supplementary Fig. 2). These serious side effects necessitated discontinuation of the ADLL (Tanganil™) treatment only a few weeks after baseline evaluation. As the treatment period was considered too short to allow for any potential disease-modifying effects, the planned 6 months follow-up assessments were not conducted (see Supplementary Results for details). Upon cessation of ADLL (Tanganil™), gait and balance symptoms improved within two weeks in two of the three patients. In parallel, the RBD phenotype reappeared at its pre-ADLL therapy level of severity (Supplementary Tables 1, 2).

Patient #3 presented with very advanced MSA-P + C at baseline and had additional severe infections with slow recovery during the follow-up period. As a result, this patient’s self-reported data could not be reliably analysed. Patient # 2 was institutionalized at the beginning of our clinical follow-up period, precluding consistent self-reporting and observation by a constant external observer. Only self-reported data from Patient #1, with the assistance of his wife (a board-qualified nurse), could be considered reliable for analysis.

UMSARS scores at neurological evaluation and weekly self-assessed by the patients, remained largely unchanged throughout the follow-up period (see Supplementary Results for details). It is important to note that the UMSARS is designed for physician-administered evaluations and may not be well-suited for self-assessment. Furthermore, its scoring system—ranging from 0 (absence of symptoms) to 4 (most severe symptoms)—may lack the sensitivity required to detect subtle, yet clinically significant changes. These limitations likely contributed to the lack of utility of the self-ratings during the follow-up. Additionally, the severity of the patients’ disease made regular follow-up visits at the Department of Neurology at UMR impractical.

### Patient #1

The patient is a 71-year-old retired business administrator whose treatment regimen consisted of duloxetine, ramipril, allopurinol, prucalopride, and a nasal corticosteroid, along with physiotherapy and occupational therapy at home.

The patient’s symptoms first emerged at age 65, beginning with instability while walking, followed by slurred speech. Initially, these symptoms were thought to be age-related. However, as the instability worsened rapidly and his speech further deteriorated, the symptoms became socially debilitating. Consequently, three years later, at age 68, he was diagnosed with clinically established MSA-C and had also developed RBD.

Given his condition, he now uses a wheelchair when outside his home and a rollator indoors. As his other MSA symptoms dominated, sleep disturbances became less of a concern. Four weeks after beginning of ADLL (Tanganil™) therapy, he noticed improvements in sleep quality and a calmer sleep pattern. His wife reported no longer hearing him shout during night sleep. However, concurrently, after two to three weeks on ADLL (Tanganil™) 5 g daily – approximately four weeks after treatment initiation- he lost the ability to stand unassisted and could no longer walk, even with the guidance of his wife, who is a nurse. He experienced repeated falls, collapsing to the floor without warning and with a high risk to injure himself, and was unable to get up without emergency assistance. Consequently, he discontinued ADLL (Tanganil™) and opted not to restart the treatment. Following discontinuation, RBD symptoms worsened, while gait gradually improved over two weeks, returning to pre-treatment levels.

### Patient #2

The patient is a 56-year-old engineer. He began experiencing symptoms of MSA at the age of 49, initially presenting with balance disturbances. He noticed increasing “clumsiness”, which progressively worsened. By age 53, and following extensive assessments, he was diagnosed with clinically established MSA-C. His condition has since deteriorated, leading to a loss of autonomy and requiring institutional care. For the past two years, he has also required a urinary catheter. His further medical history consisted of a thyroidectomy. At the start of the observational period with ADLL therapy, the patient was undergoing twice-weekly physiotherapy, speech therapy, and weekly occupational therapy. His medication regimen included baclofen to reduce nocturnal cramps, pramipexole 3.75 mg extended-release, and levodopa 100 mg extended-release in the evening. He had developed restless legs syndrome around age 50, which was alleviated with pramipexole and levodopa. Due to the severity of his other symptoms and the loss of autonomy, the patient considered his RBD symptoms to be of lesser concern. However, he had adapted his environment to protect himself during violent RBD episodes, which occurred nightly, with falls out of bed approximately once a week. Over the past 2–3 years, he had noticed a decrease in the severity of his RBD symptoms, although he still considered himself at risk of falling in the absence for protective measures.

Four weeks after initiating ADLL (Tanganil™) therapy, the patient experienced worsening balance, resulting in a fall and marked exacerbation of his pre-existing Pisa syndrome with a pronounced sensation of his trunk being strongly pulled to the right. Unlike patient #1, he did not report sudden, snap-like falls of similar intensity. The worsening of his impaired balance and of his Pisa syndrome necessitated the discontinuation of ADLL (Tanganil™). Interestingly, his RBD symptoms had nearly completely regressed within the first four weeks of therapy. Two weeks after stopping ADLL (Tanganil™), his balance gradually improved, but the RBD symptoms worsened again. To determine if the worsening was due to ADLL (Tanganil™), the therapy was reintroduced at a lower dose one month later. However, after three days of taking 3 tablets of 500 mg daily, his balance issues resurfaced, leading to the final decision to discontinue ADLL (Tanganil™). Two weeks after completely stopping ADLL-therapy, his balance returned to its prior state.

### Patient #3

The patient is a 46-year-old teacher diagnosed with clinically established MSA-P + C. Diagnosis was done only three years earlier, as for the first six years of disease progression, the diagnosis was incorrectly attributed to a psychosomatic cause. He has no significant medical history. His first symptoms appeared at age 37, beginning with erectile dysfunction. By age 40, he reported bladder dysfunction, necessitating the use of a urinary catheter, and then progressively developed an extrapyramidal syndrome. These symptoms became severely disabling, eventually leading to the loss of his ability to walk. Additionally, he frequently suffered from recurrent infections, particularly pulmonary and urinary, which each time exacerbated his MSA symptoms. When he presented to our study, he had advanced-stage MSA, was confined to a wheelchair, and had been institutionalised due to recurrent infections and worsening of MSA symptoms. Upon starting ADLL therapy, the patient also developed a severe pulmonary infection. His RBD symptoms began at age 39 and were particularly pronounced during the two years following their onset. By the time of the study, he no longer noticed RBD symptoms, although he reported that his sleep was insufficiently restorative. With ADLL therapy, he reported sudden awakenings about three hours following sleep onset, feeling as if his body was preparing to start the day. After falling back asleep, he had a particularly calm and deep sleep, with fewer dreams and difficulty waking up. However, two weeks following ADLL instauration, his trunk stability deteriorated, and his existing Pisa syndrome worsened significantly. He could no longer sit upright on the edge of the bed, as his trunk was pulled forward and to the left. He also reported episodes of severe rigidity while on ADLL, rendering him unable to move and requiring assistance on two occasions. The discontinuation of ADLL led to an improvement in symptoms, returning them to baseline levels, and the patient’s sleep patterns reverted to their previous state. (see Supplementary Results for details). During the observation period, he first developed severe pulmonary, followed by urinary, infections with a prolonged recovery over several weeks. Therefore, it remains unclear whether his truncal ataxia was attributable to ADLL (Tanganil™) therapy, or whether it was related to his concurrent infections with slow recovery.

## Discussion

In our observational case description, the three patients described a marked worsening of gait ataxia 4 to 5 weeks after treatment initiation, leading to sudden falls with a high risk of injury. This adverse effect necessitated the discontinuation of ADLL (Tanganil™).

The present observation is in contrast to the experience with ADLL in the ALCAT double-blind, randomized, placebo-controlled trial: the racemate ADLL (Tanganil™) had a favourable safety profile in patients with cerebellar ataxia excluding MSA [6]. This 108-person multicenter, clinical crossover trial was negative in respect to cerebellar symptoms, thus did not show any effect versus placebo on the primary or secondary endpoints [6]. However, caution is needed when comparing between results seen in general ataxia populations versus those specific to MSA, as ataxias are a heterogenous group of disorders with different underlying etiologies and pathophysiologies, and findings from one subgroup may not translate directly to another.

As outlined in the background section above, out of the racemate of Acetyl-DL-leucine (ADLL; Tanganil™) the L-enantiomer ALL is the bioactive, efficacious isomer of the racemate, which enters enzyme-controlled pathways that correct metabolic dysfunction and improves energy adenosine triphosphate (ATP) production [13, 16]. On the other hand, the D-enantiomer is considered to be inactive. It has been shown in *in vitro* experiments to impair the uptake of the L-enantiomer when administered in the racemate and may partially inhibit the efficacy of the active L-enantiomer [17, 18]. Whether this is the case in patients, is unknown. Furthermore, the L-enantiomer has been demonstrated to be safe and efficacious for a range of neurodegenerative diseases, that feature cerebellar ataxia, in multiple clinical trials. For example, in the recently published phase 3 trial in NPC, ALL treatment led to a statistically significant and clinically meaningful improvement of the SARA, the predefined primary endpoint, as well as all secondary endpoints [5]. The L-enantiomer demonstrated a favourable safety profile in two phase 2 trials and the phase 3 trial in NPC. None of the studies with the L-enantiomer reported any serious adverse events and the frequency of the adverse events was not different between the treatment and placebo-groups [5]. Namely, none of these studies reported adverse effects such as worsening gait ataxia or rigidity.

We think that the observed worsening of gait ataxia under ADLL (Tanganil™) is a very rare phenomenon, as it has not been reported in the literature. This finding also contrasts with the general lack of significant side effects reported in most other studies, including up to 300 reported patients treated with ADLL or ALL. One possible explanation is that this adverse effect may be specific to patients with MSA in a very advanced stage of the disease with advanced neurodegeneration. This is one of the main reasons for reporting these cases in order to raise awareness within the medical community about this rare adverse event.

Although less relevant for the participating MSA patients, we observed benefits in managing RBD in two patients with advanced MSA-C. In a previous case study, two patients with isolated RBD treated over 18 months with ADLL (Tanganil™) showed beneficial effect and markedly reduced severity and frequency of RBD symptoms within four to six weeks. This decrease persisted throughout the study period of more than two years without a loss of efficacy. Additionally, ADLL (Tanganil™) treatment reversed the loss of striatal dopamine-transporter binding and stabilised pathological metabolic brain patterns in FDG-PET, suggesting potential disease-modifying effects [3] (see Supplementary Discussion for details).

In view of the experimental and clinical results, it is surprising and remarkable, that the therapy with ADLL impaired the clinical status of the three advanced MSA patients. No data from the literature supports the hypothesis of a harmful effect of the D-enantiomer in a neurodegenerated cerebellum (See Supplemental material for details). This is also in contrast to our clinical data from a previous two-year trial in two patients with isolated RBD, who showed neither a decline in ADLL efficacy nor evidence of deleterious effects, but rather indications of a neuroprotective role in early-stage neurodegeneration [3]. It should therefore be considered that the clinical worsening of the three MSA patients under ADLL treatment could be related to the distinct pathophysiological features of MSA, especially the oligodendroglial α-synucleinopathy [19, 20]. It is conceivable that ADLL might act differently on α-synuclein harboring neurons compared to oligodendroglia, suggesting that impaired gait could be a side effect specific to MSA patients.

### Limitations of the present case descriptions

A major limitation of these case descriptions is the small sample size, with three patients, all of whom were in advanced stages of the disease, and only one patient was reliably evaluated by an external evaluator. Although our patients presented with clinically evident RBD as assessed by the clinicians with input from the spouses, no formal sleep study was performed to confirm the diagnosis. This represents a limitation, as RBD identified through clinical assessment or self-report has a sensitivity of approximately 70–90% compared with polysomnographic confirmation [21]. An additional limitation is that daily follow-up of RBD severity was based on a non-validated scale. An additional limitation is the absence of gait or stability quantification tools that were sufficiently sensitive and reliable to detect objective changes. The observational period was prematurely shortened to less than three months due to the clinical worsening of gait ataxia and balance observed in the patients. Further, the racemate, Tanganil™, was used and not the bioactive enantiomer acetyl-L-Leucine. Additionally, these case presentations had an unstructured design, as baseline and final evaluations could not be performed as planned for all three patients at the UMR due to geographical distances and the patients’ advanced disease stage and fragile health.

## Conclusions

These results are not generalizable. While ADLL alleviated RBD symptoms in our patients, neurologists should be particularly cautious, when treating patients with advanced-stage MSA with ADLL, as it may not be well tolerated and may exacerbate gait ataxia in this population. This may represent a relevant risk signal. Further studies may be conducted to explore ADLL’s effect on early stage MSA.

## Supplementary Information

Supplementary Material 1.

## Data Availability

Data are available upon reasonable request.

## Abbreviations

ADLL: N-acetyl-DL-leucine
ALL: N-acetyl-L-leucine
ATP: adenosine triphosphate
MSA: Multiple system atrophy
MSA-C: MSA-cerebellar type
MSA-P: MSA-parkinsonian type
NPC: Niemann-Pick disease type C
PD: Parkinson disease
RBD: REM-sleep behaviour disorder
REM: Rapid eye movement
SARA: Scale for the Assessment and Rating of Ataxia
