# Supplementary material for: Acetyl-DL-leucine (Tanganil™) in three patients with advanced multiple system atrophy
Source: BMC Neurol. 2025 Oct 2;25:407. doi: 10.1186/s12883-025-04451-7 (PMC12490026; doi:10.1186/s12883-025-04451-7)
Supplement: Supplementary file 1 — Supplementary Material 1. [file 12883_2025_4451_MOESM1_ESM.docx]

Journal: BMC Neurology

**Title: Acetyl-DL-leucine (Tanganil^TM^) in three patients with advanced multiple system atrophy**

***Wolfgang H. OERTEL***^1^'***, Martin HENRICH^1,2^***'***, Elisabeth SITTIG***^1^***, Philipp T. MEYER*^3^*, Michael STRUPP***^4^***, Annette JANZEN***^1^***, Fanni F. GEIBL^1,2^***&***, Elisabeth* *RUPPERT***^1,5^&

^1^ Department of Neurology, Philipps University of Marburg, Marburg, Germany

^2^ Department of Psychiatry and Psychotherapy, Philipps University of Marburg, Marburg, Germany

^3^ Department of Nuclear Medicine, Medical Center - University of Freiburg, Faculty of Medicine, University of Freiburg, Freiburg, Germany

^4^Department of Neurology, LMU University Hospital, LMU Munich, Germany

^5^Department of Neurology, CIRCSom (International Research Center for ChronoSomnology) & Sleep Disorders Center, University Hospital of Strasbourg, University of Strasbourg, France

' shared first author

& shared last author

*Address correspondence to:* [*oertelw@med.uni-marburg.de*](mailto:oertelw@med.uni-marburg.de) *& eruppert@unistra.fr*

# **Supplementary Material**

The supplementary material includes additional details on patient assessments and outcomes in the Supplementary Methods and Results sections, along with a comprehensive case description of Patient #3 in the Supplementary Results.

**Supplementary Methods**

### **Clinical assessments**

Clinical assessments included the Unified Multiple System Atrophy Rating Scale (UMSARS) [1]. For the first two patients, the Unified Multiple System Atrophy Rating Scale (UMSARS) was assessed by a neurologist at baseline (AJ) at the Department of Neurology, University Clinic Marburg (UMR). No baseline UMSARS was available for patient # 3. For patients #2 and #3, UMSARS was evaluated at the end of the protocol, five months later (ER) at the home of the patients. No end of protocol UMSARS was available for patient # 1. AJ and ER are both neurologists with experience in movement disorders; however, no specific UMSARS training was undertaken prior to the study. Additionally, patient #1 and his spouse, completed daily self-ratings of the severity of RBD symptoms, and weekly self- ratings of the frequency of RBD symptoms. Additional clinical assessments also included the Clinical Global Impression, a quality-of-life evaluation using the EQ-5D-3L from the EuroQol Group, a Visual Analog Scale for Health State (0-100), and UMSARS Parts I-III. While many of these scales are typically designed for physician evaluation, the severity of the patients’ conditions necessitated a modified approach. Therefore, self-ratings were conducted weekly, and evaluations by the neurologist were limited to baseline and the end of the protocol. Weekly self-assessments of gait and stability were performed by patients using UMSARS items 1.7 (“Walking”) and 1.8 (“Falling”). However, the questionnaires used for the self-assessment of the symptoms, with most scores ranging from 0 to 4, might not have been sensitive enough to detect objective changes in these severely affected patients between the start of the protocol and the point of treatment discontinuation. For patients #2 and #3, who were institutionalized at the beginning of the protocol and lacked reliable external assistance for evaluation, particularly for RBD, further analysis of these data was not possible. We chose to use MSA-specific scales rather than other instruments, such as the SARA commonly applied in ataxia-related research [2], in order to minimize examination time for patients who were in very advanced disease stages.

**Supplementary Results**

### **Detailed UMSARS Scores**

At baseline, neurological evaluations revealed the following UMSARS scores: Patient #1 had a UMSARS Part I score of 28 (range: 0-48) and a UMSARS Part II score of 25 (range: 0–52). End of the protocol neurological evaluation was not performed for Patient #1. At baseline, Patient #2 had 32 on UMSARS Part I and 31 on Part II. At the end of the protocol, Patient #2’s UMSARS evaluation indicated stable scores, with both UMSARS Part I and Part II scoring 33. For Patient #3, end of protocol UMSARS Part I was 38, and Part II was 34. The UMSARS Part IV score remained consistently at 4 for all three patients throughout the study protocol.

### **Detailed case description on Patient #3**

The patient is a 46-year-old teacher who was diagnosed with clinically established MSA-P only three years earlier. For the first six years of disease progression, the diagnosis was incorrectly attributed to a psychosomatic cause. He has no significant medical history and is 1.87m tall with a stable weight of 83kg. His first symptoms appeared at age 37, beginning with erectile dysfunction. By age 40, he reported bladder dysfunction, necessitating the use of a urinary catheter, and then progressively developed an extrapyramidal syndrome. These symptoms became severely disabling, eventually leading to the loss of his ability to walk, confining him to a wheelchair. Additionally, he frequently suffered from recurrent infections, particularly pulmonary and urinary, which each time exacerbated his MSA symptoms.

At the onset of ADLL therapy, the patient was receiving twice-weekly physiotherapy and speech therapy, as well as weekly occupational therapy. His medication regimen included fludrocortisone, etilefrine, levodopa/benserazide (125 mg seven times daily and retard 100 mg extended-release twice in the evening), amantadine, opicapone, and safinamide. Upon starting ADLL therapy, the patient also developed severe pulmonary infection. His RBD symptoms began at age 39 and were particularly pronounced during the two years following their onset. By the time of the study, he had been institutionalised and no longer noticed RBD symptoms, although he reported that his sleep was insufficiently restorative. With ADLL therapy, he reported sudden awakenings about three hours following sleep onset, feeling as if his body was preparing to start the day. After falling back asleep, he had a particularly calm and deep sleep, with fewer dreams and difficulty waking up. However, two weeks following ADLL instauration, his trunk stability deteriorated, and his existing Pisa syndrome worsened significantly. He could no longer sit upright on the edge of the bed, as his trunk was pulled forward and to the left. He also reported episodes of severe rigidity while on ADLL, rendering him unable to move and requiring assistance on two occasions. The discontinuation of ADLL led to an improvement in symptoms, returning them to baseline levels, and the patient's sleep patterns reverted to their previous state.

**Supplementary Discussion**

### **Detailed pathophysiological hypothesis for underlying mechanisms of observed ADLL effects**

In respect to RBD, this parasomnia is considered to be associated with a dysfunction of the coeruleus/subcoeruleus complex. In alpha-synucleinopathies aggregates of alpha-synuclein are found in noradrenergic and non-adrenergic neurons located in this upper brain stem region. In a recent still unpublished article – as only available on a preprint server - Song et al (submitted) investigated the effect of ALL in vitro in genetic PD-patient-PSC-derived dopaminergic neurons harboring GBA1 or LRRK2 mutations [3]. When these particular dopaminergic neurons were treated with Acetyl-L-Leucine, lysosomal, mitochondrial, and synaptic proteins were upregulated. ALL also upregulated expression of wild-type parkin in both GBA1 and LRRK2 mutant neurons, leading to an increase in functional dopamine transporter and synaptic membrane-associated synaptojanin-1, suggesting improved synaptic function. Finally, ALL also reduced levels of pathological pS129-alpha-synuclein. This decrease in pS129-syn was dependent on the serine protease HTRA1 which in turn was induced by ALL treatment of the dopaminergic neurons in vitro. If independently confirmed, this later finding may potentially be most relevant, as serine protease HTRA1 has been reported to disaggregate amyloid fibrils of alpha-synuclein [4]. In view of these experimental results, it is surprising and remarkable, that the therapy with ALL impaired the clinical status of the three MSA patients.

### **No data from the literature supports the hypothesis of a neurotoxic effect of the D-enantiomer in a neurodegenerated cerebellum**

The following evidence argues against a neurotoxic effect of the D-enantiomer in a neurodegenerated cerebellum: (i) Human studies: Use of ADLL in patients with degenerative cerebellar ataxias demonstrated improvement in cerebellar symptoms without reported adverse effects. Patients improved in 6 of 8 SARA subscales, and interestingly, gait also improved. [5,6]. In a recent case report of an MSA-C patient, an improvement in dysarthria was observed [7]. (ii) Animal studies of Niemann-Pick type C (NPC): A study comparing ADLL, acetyl-D-leucine, and acetyl-L-leucine in NPC mice found that all three compounds improved ataxia symptoms, indicating a symptomatic effect. However, only the racemate (ADLL) and the L-enantiomer (ALL) showed neuroprotective effects. Administration of acetyl-D-leucine alone had no neuroprotective effect but also did not worsen symptoms or indicate neurotoxicity [8]. (iii) Vestibular lesion models: In rats and cats with unilateral labyrinthectomy, ADLL and acetyl-L-leucine improved postural compensation, while acetyl-D-leucine showed no improvement. Importantly, no worsening of symptoms or neurotoxic effect was observed in these studies. Authors concluded that acetyl-D-leucine is likely inactive rather than harmful [9,10]. (iv) Sandhoff disease model: In a mouse model of Sandhoff disease, ADLL led to a moderate but significant extension of lifespan, accompanied by improved motor function and reduced glycosphingolipid storage in both the cerebrum and cerebellum. No neurotoxic effect was reported [11]. (v) Ataxia-telangiectasia: ADLL was also administered to six patients with ataxia-telangiectasia, where symptomatic improvement was documented. No neurotoxic effects were reported [12].

It should be noted that in MSA patients, the ataxic symptoms and RBD have different pathophysiological mechanisms. Most investigations of ADLL and ALL have focused on neuronal effects and synuclein aggregation in neurons. In MSA, however, α-synuclein accumulates primarily in oligodendroglial cells. Currently, no data exist on the effects of ADLL on diseased oligodendroglia, and neither ADLL nor ALL has been tested in an MSA animal model. This highlights the need for further studies to clarify the impact of these compounds on glial pathology.

**Supplementary Tables**

### ***Supplementary Table 1***

Title Supplementary table 1:

**Summary table of main clinical characteristics and results:**

|  | **Patient 1** | **Patient 2** | **Patient 3** |
| --- | --- | --- | --- |
| Age (years) | 71 | 56 | 46 |
| Age of onset | 65 | 49 | 37 |
| MSA subtype | MSA-C | MSA-C | MSA-P+C |
| Severity of RBD (0-4) | 2 | 2 | 0 |
| Response to ADLL  - RBD  - Ataxia | - Improved  - Worsening of gait, resulting in inability to use a walker | - Improved  - Worsening of PISA syndrome; unable to maintain a seated position | - NA  - Difficult to evaluate due to concurrent infectious diseases; worsening of PISA syndrome; became bedridden |
| UMSARS scores:  Baseline  UMSARS Part I (0-48)  UMSARS Part II (0-52)  UMSARS Part IV (0-5)  Final evaluation  UMSARS Part I  UMSARS Part II  UMSARS Part IV | 28  25  4  NA  NA  NA | 32  31  4  33  33  4 | NA  NA  NA  38  34  4 |
| RBDSQ [13] | 9 | 12 | NA |
| Adverse effects | Snap-like falls and loss of ambulation (progression from walking with a walker to being wheelchair-bound) | Worsening of PISA syndrome with major difficulty maintaining a seated position | Worsening of PISA syndrome; unable to sit and became bedridden, concomitant with infectious diseases |
| Reversibility | Complete | Complete | Difficult to evaluate due to concurrent infectious diseases; reversibility was delayed but appeared complete |

### ***Supplementary Table 2***

Title Supplementary table 2:

**Summary table of chronological description for each patient:**

|  | **Patient 1** | **Patient 2** | **Patient 3** |
| --- | --- | --- | --- |
| Baseline | Day -1 | Day -1 | Day -1 |
| Treatment initiation / ADLL uptitration | Day 1 to 9 | Day 1 to 9 | Day 1 to 9 |
| Weeks 2-3:  ADLL full dosage (days) | Day 10 | Day 10 | Day 10 |
| RBD improvement | About day 29 | About day 29 | NA |
| Week 4:  ADLL discontinuation due to adverse events | Day 39 | Day 42 | Day 39 |
| Post-discontinuation:  Complete recovery | 2 weeks after stopping ADLL | 2 weeks after stopping ADLL | About 6 weeks after stopping ADLL; biased by concurrent infectious diseases |
| ADLL reintroduction | NA | Day 70 | NA |
| ADLL discontinuation | NA | Day 73 | NA |
| Complete recovery | NA | 2 weeks after stopping ADLL | NA |

**Supplementary Figures**

### ***Supplementary Figure 1***

Title Supplementary Figure 1:

Daily evolution of REM sleep behavior (RBD) severity in Patient #1 over the study period.


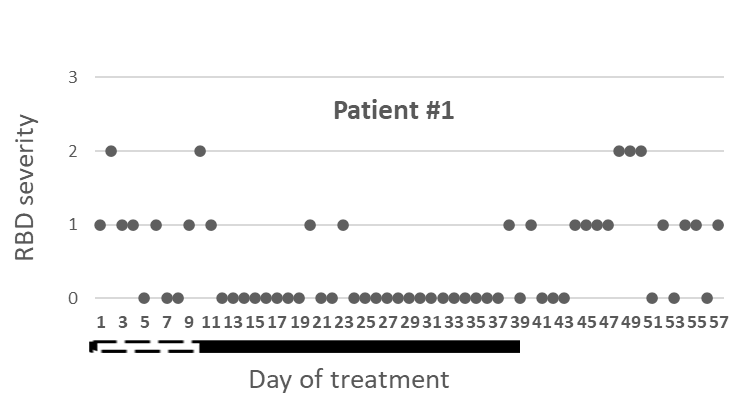


Legend Supplementary Figure 1:

Patient # 1, with the assistance of his wife, assessed the severity of RBD symptoms daily throughout the study period using the following scale: 0 = no RBD symptoms as documented by patient and partner; 1 = speaking and/or slight movements (jerks); 2 = screaming, shouting or complex, non-aggressive movements; 3 = complex movements with risk of injury of herself/himself or partner; 4 = movements so severe that subject falls out of bed. The treatment period is indicated by a solid black bar, with the dose uptitration phase indicated by a dotted line.

### ***Supplementary Figure 2***

Title Supplementary Figure 2:

Weekly assessment of overall health state severity in Patient #1.


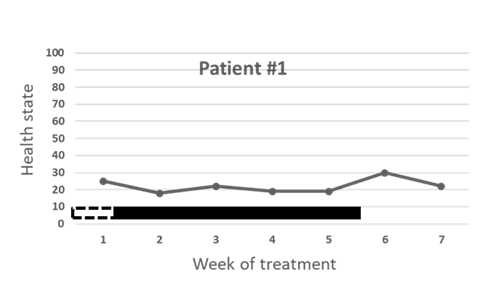


Legend Supplementary Figure 2:

Patient #1, with the assistance of his wife, assessed his health state using a visual analogue scale, where 100 represented the best imaginable state and 0 the worst. The treatment period is indicated by a solid black bar, with the dose uptitration phase indicated by a dotted line.

**Supplementary References**

1. Wenning GK, Tison F, Seppi K, Sampaio C, Diem A, Yekhlef F, et al. Development and validation of the Unified Multiple System Atrophy Rating Scale (UMSARS). Movement Disorders. 2004 Dec;19(12):1391–402.

2. Schmitz-Hübsch T, du Montcel ST, Baliko L, Berciano J, Boesch S, Depondt C, et al. Scale for the assessment and rating of ataxia: development of a new clinical scale. Neurology. 2006 Jun 13;66(11):1717–20.

3. Song P, Franchini R, Chen C, Duong B, Wang YZ, Savas J, et al. N-acetyl-l-leucine lowers pS129-synuclein and improves synaptic function in models of Parkinson’s disease. Res Sq. 2025 Apr 9;rs.3.rs-6298077.

4. Chen S, Puri A, Bell B, Fritsche J, Palacios HH, Balch M, et al. HTRA1 disaggregates α-synuclein amyloid fibrils and converts them into non-toxic and seeding incompetent species. Nat Commun. 2024 Mar 18;15(1):2436.

5. Strupp M, Teufel J, Habs M, Feuerecker R, Muth C, van de Warrenburg BP, et al. Effects of acetyl-dl-leucine in patients with cerebellar ataxia: a case series. J Neurol. 2013 Oct;260(10):2556–61.

6. Becker-Bense S, Kaiser L, Becker R, Feil K, Muth C, Albert NL, et al. Acetyl-DL-leucine in cerebellar ataxia ([18F]-FDG-PET study): how does a cerebellar disorder influence cortical sensorimotor networks? J Neurol. 2023 Jan;270(1):44–56.

7. Lehner L, Strupp M. Acetyl-Leucine Improves Cerebellar Dysarthria in MSA-C. Mov Disord Clin Pract. 2025 Aug 25;

8. Kaya E, Smith DA, Smith C, Morris L, Bremova-Ertl T, Cortina-Borja M, et al. Acetyl-leucine slows disease progression in lysosomal storage disorders. Brain Commun. 2021;3(1):fcaa148.

9. Tighilet B, Leonard J, Bernard-Demanze L, Lacour M. Comparative analysis of pharmacological treatments with N-acetyl-DL-leucine (Tanganil) and its two isomers (N-acetyl-L-leucine and N-acetyl-D-leucine) on vestibular compensation: Behavioral investigation in the cat. Eur J Pharmacol. 2015 Dec 15;769:342–9.

10. Zwergal A, Günther L, Beck R, Xiong G, Brandt T, Jahn K, et al. N-Acetyl-L-leucine beschleunigt die vestibuläre Kompensation durch Modulation cerebellärer und thalamischer Plastizität. Klinische Neurophysiologie. 2014 Mar 12;45:V11.

11. Kaya E, Smith DA, Smith C, Boland B, Strupp M, Platt FM. Beneficial Effects of Acetyl-DL-Leucine (ADLL) in a Mouse Model of Sandhoff Disease. J Clin Med. 2020 Apr 8;9(4):1050.

12. Brueggemann A, Bicvic A, Goeldlin M, Kalla R, Kerkeni H, Mantokoudis G, et al. Effects of Acetyl-DL-Leucine on Ataxia and Downbeat-Nystagmus in Six Patients With Ataxia Telangiectasia. J Child Neurol. 2022 Jan;37(1):20–7.

13. Stiasny‐Kolster K, Mayer G, Schäfer S, Möller JC, Heinzel‐Gutenbrunner M, Oertel WH. The REM sleep behavior disorder screening questionnaire—A new diagnostic instrument. Movement Disorders. 2007 Dec 15;22(16):2386–93.
